# Serum SERPINA3 as a Candidate Non-Invasive Biomarker for Neuromyelitis Optica Spectrum Disorder: Proteomic Discovery and Same-Center Validation

**DOI:** 10.3390/diagnostics16162660

**Published:** 2026-08-20

**Authors:** Ting Xu, Bingqing Han, Guanghui Zheng, Wencan Jiang, Hanyu Zhang, Guojun Zhang

**Affiliations:** 1 Department of Clinical Diagnosis, Laboratory of Beijing Tiantan Hospital, Capital Medical University, Beijing 100070, China; 2 Laboratory Diagnosis Platform for Nervous System Infectious Diseases, Laboratory for Clinical Medicine, Capital Medical University, Beijing 100070, China; 3 Beijing Key Laboratory of Biomarker Screening and Translational Research in Neurological Diseases, Beijing 100070, China; 4 The Fifth Clinical Medical School, Capital Medical University, Beijing 100070, China

**Keywords:** neuromyelitis optica spectrum disorder, SERPINA3, routine hematology, candidate biomarker

## Abstract

**Background:** Diagnostic evaluation of suspected neuromyelitis optica spectrum disorder (NMOSD) integrates AQP4-IgG testing, clinical assessment, neuroimaging, and exclusion of alternative inflammatory demyelinating disorders; nevertheless, some patients remain diagnostically unresolved or may be misclassified as multiple sclerosis (MS) or other disorders. **Methods:** This single-center retrospective exploratory study analyzed quantitative serum proteomics from 20 patients with MS and 20 with NMOSD. Proteins meeting nominal *p* < 0.05 together with prespecified fold-change criteria were considered exploratory candidates. VWF, PPBP, and SERPINA3 were subsequently assessed by ELISA in a separate, non-overlapping cohort from the same center, together with routine hematological variables. **Results:** Among 261 protein entries, 34 met the exploratory nominal threshold and fold-change criteria, but none remained significant after Benjamini–Hochberg correction (lowest q = 0.0734). VWF and PPBP showed no significant differences across the four validation groups. SERPINA3 differed across groups (Kruskal–Wallis H = 44.982, *p* = 9.34 × 10^−10^) and was higher in NMOSD than in MS, TBI, and HCs after Holm adjustment (adjusted *p* = 0.036, 1.49 × 10^−9^, and 1.13 × 10^−5^, respectively). In the full ELISA cohort, the AUC was 0.928 for NMOSD versus HC and 0.800 for NMOSD versus MS. In a post hoc sensitivity analysis excluding participants with preceding infection, the NMOSD–MS separation was attenuated. In smaller complete-case analyses, adding an additional laboratory variable to SERPINA3 did not significantly improve apparent discrimination. **Conclusions:** SERPINA3 is therefore an exploratory adjunctive serum biomarker candidate rather than a stand-alone diagnostic test; prospective external validation in clinically representative cohorts is required.

## 1. Introduction

Neuromyelitis optica spectrum disorder (NMOSD) is a severe autoimmune inflammatory disorder of the central nervous system (CNS) that predominantly affects the optic nerves and spinal cord but may also involve other characteristic CNS regions [1]. NMOSD is now recognized as an autoimmune astrocytopathy characterized by astrocytic injury and a pathobiology distinct from that of multiple sclerosis (MS) [2]. Nevertheless, overlapping clinical and imaging features can make the differentiation of NMOSD from MS and other inflammatory demyelinating disorders challenging in routine practice [3].

Accurate differentiation is clinically important because the therapeutic strategies and long-term prognoses of NMOSD and MS differ substantially. High-dose glucocorticoids and plasma exchange are commonly used for acute attacks of NMOSD, followed by long-term immunosuppressive therapy to prevent relapse [4]. In contrast, some disease-modifying therapies used for MS, including interferon-β and fingolimod, may exacerbate disease activity or neurological impairment in patients with NMOSD [5,6]. Early and accurate differentiation is therefore essential to guide appropriate treatment and reduce the risk of disability accumulation.

AQP4-IgG is the key serological biomarker for NMOSD, but a clinically important proportion of patients are seronegative, which can complicate laboratory-supported diagnosis [7]. Cerebrospinal fluid oligoclonal bands (OCBs) are an important laboratory marker for MS and are incorporated into its diagnostic evaluation; however, OCBs may also occur in NMOSD and therefore cannot independently distinguish NMOSD from MS [8,9]. Existing laboratory indicators and imaging examinations may still be insufficient for accurate diagnosis in some patients, while lumbar puncture is invasive and unsuitable for frequent monitoring. These limitations highlight the need for non-invasive and readily measurable serum biomarkers that could complement established diagnostic evaluation.

Proteomic technologies provide a powerful approach for screening candidate biomarkers in neuroinflammatory diseases [10,11], with wide applications in cerebrospinal fluid (CSF) [12,13,14,15,16], serum [17], and urine [18]. Given the accessibility of serum for repeated testing, serum proteomics may facilitate the identification of candidate biomarkers with potential clinical utility. In the present study, we performed TMT-based serum proteomic screening followed by ELISA assessment in a separate cohort to identify and evaluate candidate serum proteins, with particular focus on SERPINA3, and to explore their potential value in supporting the diagnosis of NMOSD and its differentiation from MS.

## 2. Materials and Methods

### 2.1. Study Participants

This single-center retrospective observational study was conducted at Beijing Tiantan Hospital, Capital Medical University. A total of 118 eligible adults were included from clinical records and archived serum collections. The discovery cohort comprised 40 patients treated between January 2018 and December 2020, including 20 patients with NMOSD and 20 with MS. The separate, non-overlapping same-center validation cohort comprised 78 participants recruited between January 2021 and December 2022, including 19 patients with NMOSD, 20 with MS, 20 with traumatic brain injury (TBI), and 19 healthy controls (HCs).

The discovery cohort was designed as an exploratory, balanced proteomic set. Eligible archived serum samples were selected using the same group-specific eligibility and exclusion criteria applied to the validation cohort. All 40 discovery samples underwent TMT labeling and LC–MS/MS analysis and were included in the participant-level proteomic analysis. The participant-selection process is summarized in Figure S1.

All participants were aged 18 years or older and were required to meet the corresponding group-specific criteria described below. Serum samples had to be of sufficient volume and acceptable analytical quality, with adequate source documentation to verify the diagnosis and relevant clinical information, including sampling time, disease phase, treatment exposure, infection status, and core clinical characteristics.

Patients in the NMOSD group fulfilled the 2015 International Panel for NMO Diagnosis consensus criteria [19], whereas patients in the MS group fulfilled the 2017 revised McDonald criteria [20], which were the applicable criteria during recruitment in 2018–2022. In both groups, serum samples were collected before the initiation of acute-phase immunotherapy and in the absence of active infection at blood collection. Disease phase, treatment exposure, infection status, and AQP4-IgG status were retrieved from the medical records.

Active infection was defined as an ongoing infection documented by the treating physician and supported by at least one of the following: initiation or continuation of systemic anti-infective treatment, a positive microbiological test, imaging findings compatible with infection, or clinical and laboratory findings considered consistent with active infection. A preceding infection was defined as a clinically documented infection occurring within the 4 weeks before the neurological attack but considered resolved at the time of serum collection. Acute-phase sampling was defined as blood collection within 7 days after the onset of a new or worsening neurological episode attributed to relapse.

The TBI group included patients with acute traumatic brain injury independently confirmed by two experienced neurosurgeons on the basis of a documented trauma history, serial Glasgow Coma Scale assessments, and cranial computed tomography findings. Serum samples were collected as soon as feasible after hospital admission. Patients were excluded from the TBI group if they had a known inflammatory demyelinating disease, another active autoimmune or inflammatory neurological disorder, or clinically documented active infection at the time of blood collection.

HCs were adults without a known history of neurological disease, systemic autoimmune disease, active malignancy, or chronic inflammatory disease. Individuals were excluded if, within the 4 weeks before blood collection, they had experienced a clinically apparent infection, received systemic anti-infective treatment, undergone major surgery, or sustained major trauma.

Common exclusion criteria for all groups were an unconfirmed diagnosis or inability to reliably link the serum sample to the corresponding clinical record, an established alternative inflammatory demyelinating disorder where applicable, severe cardiac, hepatic, renal, or other major organ dysfunction, active malignancy, another active systemic autoimmune disease, active infection at the time of sampling, severe hemolysis or lipemia, inadequate serum volume, or improper sample storage.

This study involved the retrospective use of archived serum samples and corresponding clinical data. The study protocol was approved by the Institutional Review Board of Beijing Tiantan Hospital, Capital Medical University (No. KY 2026-091-02), and the study was conducted in accordance with the Declaration of Helsinki. Written informed consent for sample collection and research use was obtained from all participants at the time of the original blood collection.

### 2.2. Sample Collection

Residual serum specimens (approximately 2–3 mL) from routine clinical testing were used. After centrifugation at 3000× *g* for 10 min at room temperature, serum was aliquoted into 1.5 mL EP tubes immediately and stored at −80 °C. All samples were limited to only one freeze-thaw cycle before subsequent proteomic and ELISA detection to avoid protein degradation.

### 2.3. Laboratory Parameter Measurement

At the same venipuncture, separate blood specimens were collected for routine hematological and clinical chemistry testing. Residual serum remaining after clinical chemistry testing was stored in the institutional biobank and subsequently used for the protein assays in this study. Routine hematological parameters included total white blood cell count (WBC); absolute and percentage counts of lymphocytes, monocytes, neutrophils, eosinophils, and basophils; red blood cell count (RBC); hemoglobin (HGB); hematocrit (HCT); mean corpuscular volume (MCV); mean corpuscular hemoglobin (MCH); mean corpuscular hemoglobin concentration (MCHC); red cell distribution width (RDW); platelet count (PLT); platelet distribution width (PDW); mean platelet volume (MPV); platelet large-cell ratio (P-LCR); and plateletcrit (PCT). All hematological measurements were performed using a Mindray BC-6900 automated hematology analyzer under routine internal quality-control procedures.

### 2.4. Proteomic Analysis

#### 2.4.1. Serum Protein Preparation, Digestion, and TMT Labeling

Serum protein concentrations were determined using the bicinchoninic acid assay after enrichment of low-abundance proteins using the ProteoMiner Protein Enrichment Kit (Bio-Rad, Hercules, CA, USA). For each participant, 100 μg of serum protein was subjected to enzymatic digestion using a modified filter-aided sample preparation method [21].

Briefly, each sample was mixed with 200 μL of UA buffer containing 8 M urea and 150 mM Tris–HCl, pH 8.0, transferred to a 10-kDa centrifugal filter unit, and centrifuged at 12,000× *g* for 15 min. An additional 200 μL of UA buffer was added, followed by centrifugation under the same conditions, and the filtrate was discarded. The retained proteins were alkylated by adding 100 μL of 50 mM iodoacetamide prepared in UA buffer. The filter units were mixed at 600 rpm for 1 min, incubated at room temperature in the dark for 30 min, and centrifuged at 12,000× *g* for 10 min. The filter units were then washed twice with 100 μL of UA buffer and twice with 200 μL of 100 mM triethylammonium bicarbonate, with centrifugation at 12,000× *g* for 10 min after each wash. Eight microliters of trypsin solution containing 4 μg of sequencing-grade trypsin was subsequently added to each filter unit. The samples were mixed at 600 rpm for 1 min and incubated at 37 °C for 16–18 h. The digested peptides were collected by centrifugation at 14,000× *g* for 10 min and lyophilized.

Peptides were labeled using the TMT 10-plex Label Reagent Set (Thermo Fisher Scientific, Waltham, MA, USA; catalog no. 90111) according to the manufacturer’s instructions. TMT reagents were equilibrated to room temperature for approximately 30 min and dissolved in 41 μL of acetonitrile. Each reagent was added to 100 μg of peptide sample, followed by incubation at room temperature for 1 h. The reaction was quenched with 8 μL of hydroxylamine and incubated for an additional 15 min at room temperature.

The 40 serum samples were analyzed in four TMT 10-plex batches, each containing five individual MS samples and five individual NMOSD samples. Each reporter channel represented one biological sample from one participant, and no samples were pooled before labeling. Within each batch, MS samples were labeled with TMT tags 126, 127N, 127C, 128N, and 128C, whereas NMOSD samples were labeled with 129N, 129C, 130N, 130C, and 131. No common pooled-reference channel was included because all ten channels were assigned to individual samples. After labeling and quenching, the ten individually labeled peptide samples within each batch were combined in equal amounts, desalted, and lyophilized.

#### 2.4.2. High-pH Reversed-Phase Fractionation

For each TMT batch, the combined TMT-labeled peptide mixture was fractionated separately using a UPLC 3000 system equipped with an XBridge BEH300 C18 column. A total of 48 fractions were collected and concatenated into 12 pooled fractions. The pooled fractions were vacuum-dried and reconstituted in 0.1% formic acid before LC–MS/MS analysis.

#### 2.4.3. LC–MS/MS Analysis and Technical Replicates

TMT-labeled peptide fractions were analyzed using an EASY-nLC 1000 nano-liquid chromatography system coupled to a Q Exactive mass spectrometer (Thermo Fisher Scientific). Peptides were separated on a 15-cm analytical column with an internal diameter of 75 μm, packed with ReproSil-Pur C18-AQ 3.0-μm reversed-phase material (Dr. Maisch GmbH, Ammerbuch, Germany).

Mass spectrometric data were acquired using Xcalibur software (version 4.4; Thermo Fisher Scientific, Waltham, MA, USA) in data-dependent top-15 acquisition mode. Full MS scans were acquired over an *m*/*z* range of 300–1500 at a resolution of 120,000 at *m*/*z* 200, with an automatic gain control target of 3 × 10^6^ and a maximum injection time of 50 ms.

The 15 most abundant precursor ions in each full scan were selected using an isolation window of 1.0 Da and fragmented by higher-energy collisional dissociation at a normalized collision energy of 30%. MS/MS spectra were acquired at a resolution of 60,000 at *m*/*z* 200, with an automatic gain control target of 1 × 10^5^ and a maximum injection time of 50 ms. Dynamic exclusion was set to 20 s.

Each peptide fraction was analyzed in two consecutive LC–MS/MS runs. These runs were technical replicate injections of the same TMT-labeled fraction rather than independent biological replicates. The raw files from the two technical runs were processed together using the same MaxQuant search and quantification workflow. Reporter-ion evidence obtained from the technical replicate runs was aggregated at the peptide and protein-group levels to generate a single quantitative profile for each biological sample. The two technical runs were not treated as independent observations and did not increase the biological sample size.

#### 2.4.4. Database Search, Protein Identification, and Quantification

All MS/MS spectra were processed using MaxQuant version 1.6.0.16 and searched against the UniProt human FASTA protein database released in April 2021, containing 92,607 protein entries. Trypsin was specified as the proteolytic enzyme, with up to two missed cleavages allowed. TMT 10-plex labeling of lysine residues and peptide N-termini was specified, carbamidomethylation of cysteine residues was set as a fixed modification, and oxidation of methionine residues was set as a variable modification. The precursor-ion and fragment-ion mass tolerances were set to 10 ppm and 20 ppm, respectively. Peptide-spectrum match and protein-level false discovery rates were controlled at ≤1%.

The four TMT 10-plex batches were defined as separate experiments in the MaxQuant experimental design. The corresponding high-pH fractions and technical replicate injections were assigned to their respective TMT batches, and all raw files were processed using identical database-search and quantification parameters. Technical replicate injections were combined during peptide- and protein-level quantification and were not treated as independent biological observations.

Reporter-ion quantification was based on the TMT reporter-ion signals extracted from each identified peptide. To minimize interference from precursor co-isolation, only spectra with a parent ion fraction of at least 0.75 were included in reporter-ion quantification. Protein abundance was calculated by summing the reporter-ion intensities of peptide-spectrum matches assigned to each protein group. Reporter-ion intensities were normalized under the assumption of equal protein loading across TMT channels.

Protein groups identified only by site, reverse-database matches, or potential contaminants were excluded. Protein groups were retained if they met the prespecified peptide-identification and quantitative-completeness criteria. After quality filtering, 261 protein entries across the 20 MS and 20 NMOSD samples were retained for downstream statistical analysis.

### 2.5. ELISA Validation

Serum concentrations of SERPINA3, PPBP, and VWF were measured using commercial ELISA kits according to the manufacturers’ instructions: SERPINA3 (Abcam, Waltham, MA, USA; ab157706), PPBP (Abcam, Waltham, MA, USA; ab216171), and VWF (Abcam, Waltham, MA, USA; ab223864). For each analyte, two kits were used. Samples were assigned coded identifiers, and the laboratory personnel performing the assays were blinded to the participants’ diagnostic groups.

Standards and serum samples were assayed in duplicate. Absorbance was measured at 450 nm using a microplate reader, and a separate calibration curve was generated for each plate. The mean value of the duplicate wells was interpolated from the corresponding plate-specific calibration curve and multiplied by the applicable dilution factor to obtain the final serum concentration.

For VWF measurement, serum samples were diluted 1:4000. The calculated concentrations were converted to μg/mL, and all diluted samples fell within the manufacturer-specified calibration range of 0.469–30 ng/mL.

For PPBP measurement, serum samples were initially diluted 1:4000. Samples with concentrations below the lower limit of the calibration curve were reassayed at a dilution of 1:1000.

For SERPINA3 measurement, serum samples were initially diluted 1:5000. Samples with concentrations above the upper limit of the calibration curve were reassayed at a dilution of 1:10,000.

### 2.6. Bioinformatics Analysis

For the discovery comparison, an independent two-sample Student *t* test was applied to each protein. Proteins with a fold change greater than 1.2 or less than 1/1.2 and nominal *p* < 0.05 were treated as exploratory candidates. Benjamini-Hochberg correction was applied across all 261 protein tests. For visualization, abundance values were transformed as log2 (x + 1) and standardized to row-wise z scores. Hierarchical clustering was performed for proteins and separately within each prespecified diagnostic group.

### 2.7. Statistical Analysis

Continuous variables are presented as medians with interquartile ranges, and categorical variables as counts and percentages. Baseline continuous variables were compared using the Kruskal–Wallis test or Mann–Whitney U test, as appropriate. Categorical variables were compared using Fisher’s exact test or the Fisher–Freeman–Halton exact test.

VWF and SERPINA3 were compared among the four groups using the Kruskal–Wallis test, whereas PPBP was analyzed using Welch’s analysis of variance because of unequal variances. Significant SERPINA3 results were followed by Dunn’s tests with Holm adjustment for the prespecified comparisons of NMOSD with MS, TBI, and HC.

Diagnostic performance was assessed using receiver operating characteristic curves. AUCs and 95% confidence intervals were estimated by stratified bootstrap resampling. Correlations between SERPINA3 and laboratory indicators were evaluated separately within each group using Spearman’s rank correlation, with Benjamini–Hochberg correction for multiple testing. Exploratory logistic-regression models evaluated SERPINA3 alone and SERPINA3 plus one additional laboratory variable in complete cases; missing values were not imputed. For each comparison, candidate combined models were evaluated by leave-one-out cross-validation (LOOCV), and the additional predictor with the highest LOOCV AUC was selected. Because predictor selection was performed within the same complete-case dataset rather than nested within an outer validation loop, the LOOCV AUCs were treated as exploratory internal estimates. Continuous predictors were z-standardized using the mean and standard deviation of the corresponding complete-case comparison dataset before fitting the final logistic-regression model. Correlated apparent AUCs were compared using the DeLong test.

A post hoc sensitivity analysis was performed to assess potential confounding by preceding infection. The six NMOSD and one MS participants with a clinically documented preceding infection were excluded, leaving 13 NMOSD, 19 MS, and 19 HC participants. SERPINA3 concentrations were compared across these three groups using the Kruskal–Wallis test, followed by NMOSD-focused Dunn tests with Holm adjustment. ROC AUCs for NMOSD versus MS and NMOSD versus HC were re-estimated using 10,000 stratified bootstrap resamples.

All tests were two-sided, and *p* < 0.05 was considered statistically significant unless otherwise specified. Analyses were performed using Python 3.13.5.

### 2.8. Use of Generative Artificial Intelligence

During the preparation and revision of this manuscript, ChatGPT (GPT-5.6 Sol; OpenAI, San Francisco, CA, USA) was used solely to assist with English-language editing, grammatical correction, and improvement of the clarity, readability, and organization of the manuscript. The tool was not used for study design, participant selection, generation or modification of primary data, statistical analysis, generation of figures or tables, or final scientific interpretation of the results. All AI-assisted content was critically reviewed, verified, and revised by the authors before inclusion in the manuscript.

## 3. Results

### 3.1. Demographic and Clinical Characteristics of the Study Cohorts

The discovery proteomic cohort comprised 20 patients with NMOSD and 20 with MS. Baseline characteristics are provided in Table S1, and participant flow is shown in Figure S1. Sex distribution, age at enrollment, and age at disease onset were comparable between the two groups (*p* = 1.000, 0.516, and 0.655, respectively). Median EDSS at sampling was higher in NMOSD than in MS (4.8 [IQR, 3.8–6.0] vs. 2.5 [IQR, 1.8–3.2], *p* < 0.001). Acute-phase sampling occurred in 16 of 20 patients with NMOSD (80.0%) and 17 of 20 patients with MS (85.0%; *p* = 1.000).

The non-overlapping validation cohort comprised 19 patients with NMOSD, 20 with MS, 20 with TBI, and 19 HCs. Age and sex distributions were comparable among the four groups. Patients with NMOSD and MS had similar ages at disease onset and proportions of acute-phase sampling, with 16 of 19 patients with NMOSD and 18 of 20 patients with MS sampled within the prespecified acute-phase window.

Patients with NMOSD had higher disability scores at onset than those with MS. A preceding infection was reported in six patients with NMOSD and one patient with MS. Five patients with NMOSD were AQP4-IgG seronegative, including one who was MOG-IgG positive and four who were MOG-IgG negative. Clinical characteristics are summarized in Table 1.

### 3.2. Proteomic Profiling and Candidate Biomarker Discovery

**Figure 1:**
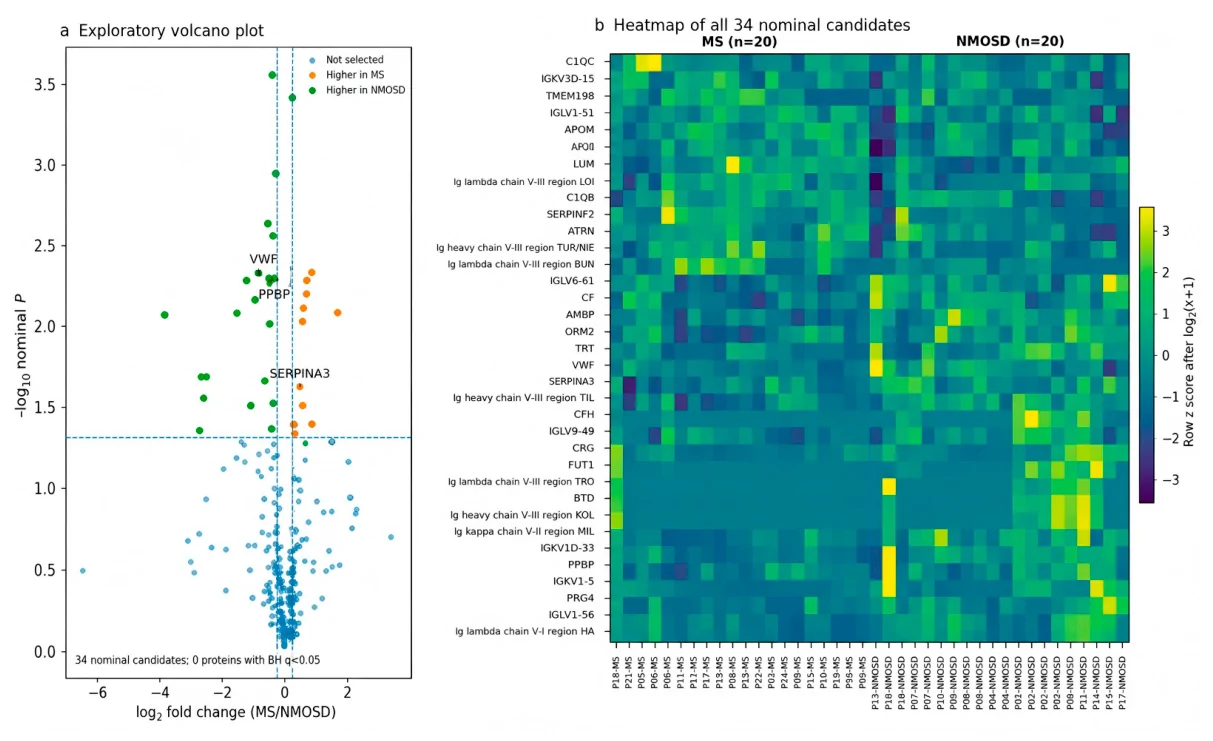
Exploratory proteomic discovery analysis in all 20 MS and 20 NMOSD samples. (**a**) Volcano plot of 261 quantified protein entries. Proteins meeting nominal *p* < 0.05 and the prespecified fold-change criteria are highlighted. (**b**) Heatmap of the 34 nominal candidates using row-wise z scores after log2 (x + 1) transformation. VWF, PPBP, and SERPINA3 are marked with a dagger. No protein met the conventional Benjamini–Hochberg q < 0.05 threshold.

Among the 261 quantified protein entries, 34 met the nominal *p* < 0.05 and prespecified fold-change criteria (Table S2), including 13 with higher abundance in MS and 21 with higher abundance in NMOSD. VWF, PPBP, and SERPINA3 were among the proteins with higher abundance in NMOSD. No protein remained significant after Benjamini–Hochberg correction, with the lowest q value being 0.0734 (Figure 1).

In the processed matrix, SERPINA3, VWF, and PPBP all showed higher mean abundance in NMOSD than in MS, with fold changes of 1.61, 1.77, and 1.97, respectively. The corresponding nominal *p* values were 0.0217, 0.0047, and 0.0067, with Benjamini–Hochberg q values of 0.2465, 0.1300, and 0.1300, respectively.

### 3.3. ELISA Validation of Candidate Biomarkers

Serum VWF levels were comparable across the NMOSD, MS, TBI, and HC groups (Kruskal–Wallis H = 0.457, *p* = 0.928). Median concentrations were 45.07 μg/mL (IQR, 26.73–69.93) in NMOSD, 50.85 μg/mL (IQR, 39.30–58.11) in MS, 52.00 μg/mL (IQR, 17.22–61.24) in TBI, and 43.17 μg/mL (IQR, 25.51–64.77) in HC. PPBP showed a similar pattern, with no significant between-group difference (Welch’s ANOVA, F = 0.416, *p* = 0.743). The corresponding median concentrations were 241.66 ng/mL (IQR, 91.05–322.17), 229.17 ng/mL (IQR, 146.83–255.89), 236.80 ng/mL (IQR, 101.81–305.51), and 258.32 ng/mL (IQR, 166.70–304.12), respectively.

**Figure 2:**
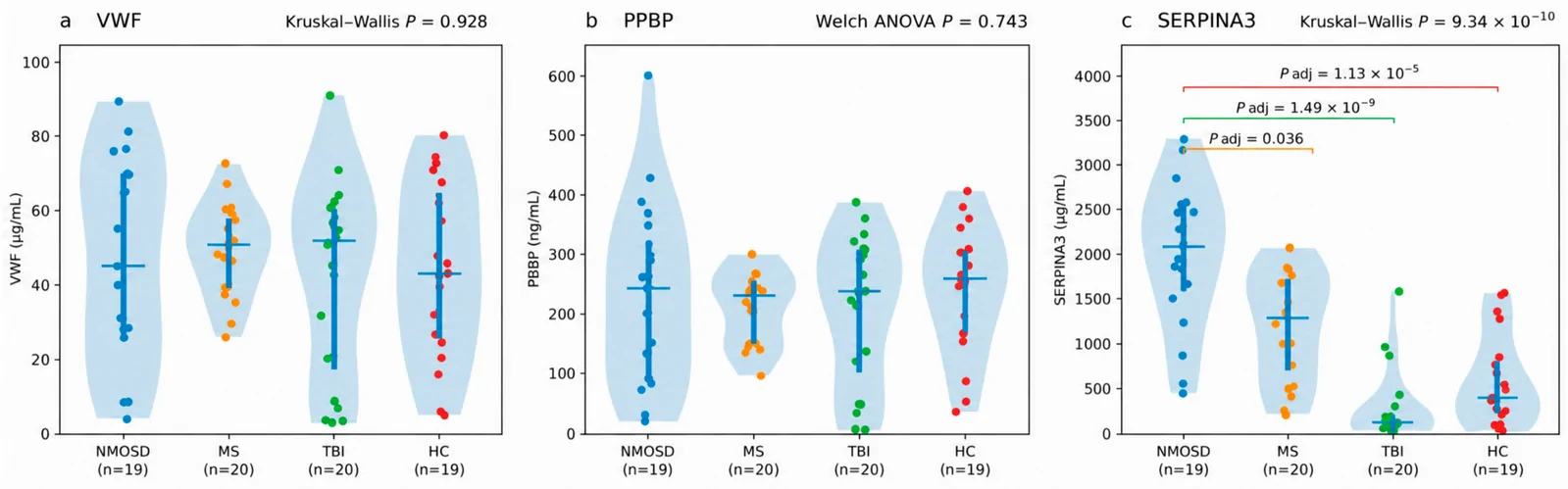
ELISA assessment of VWF, PPBP, and SERPINA3 in the validation cohort. Serum concentrations of (**a**) VWF, (**b**) PPBP, and (**c**) SERPINA3 are shown for patients with NMOSD, MS, and TBI and for HCs. Violin plots represent the distribution of the data, individual dots represent participants, horizontal lines indicate medians, and vertical bars indicate interquartile ranges. HC, healthy controls; MS, multiple sclerosis; NMOSD, neuromyelitis optica spectrum disorder; PPBP, pro-platelet basic protein; TBI, traumatic brain injury; VWF, von Willebrand factor; SERPINA3, alpha 1 Antichymotrypsin.

By contrast, SERPINA3 levels differed markedly among the four groups (Kruskal–Wallis H = 44.982, *p* = 9.34 × 10^−10^). Median SERPINA3 concentrations were highest in NMOSD at 2092.64 μg/mL (IQR, 1581.95–2558.96), followed by MS at 1288.33 μg/mL (IQR, 707.38–1731.74), HC at 399.17 μg/mL (IQR, 227.11–807.11), and TBI at 128.53 μg/mL (IQR, 75.29–223.43). In the three prespecified NMOSD-focused post hoc comparisons, SERPINA3 concentrations were significantly higher in NMOSD than in MS, TBI, and HC, with Holm-adjusted *p* values of 0.036, 1.49 × 10^−9^, and 1.13 × 10^−5^, respectively (Figure 2).

### 3.4. Diagnostic Performance of SERPINA3

**Figure 3:**
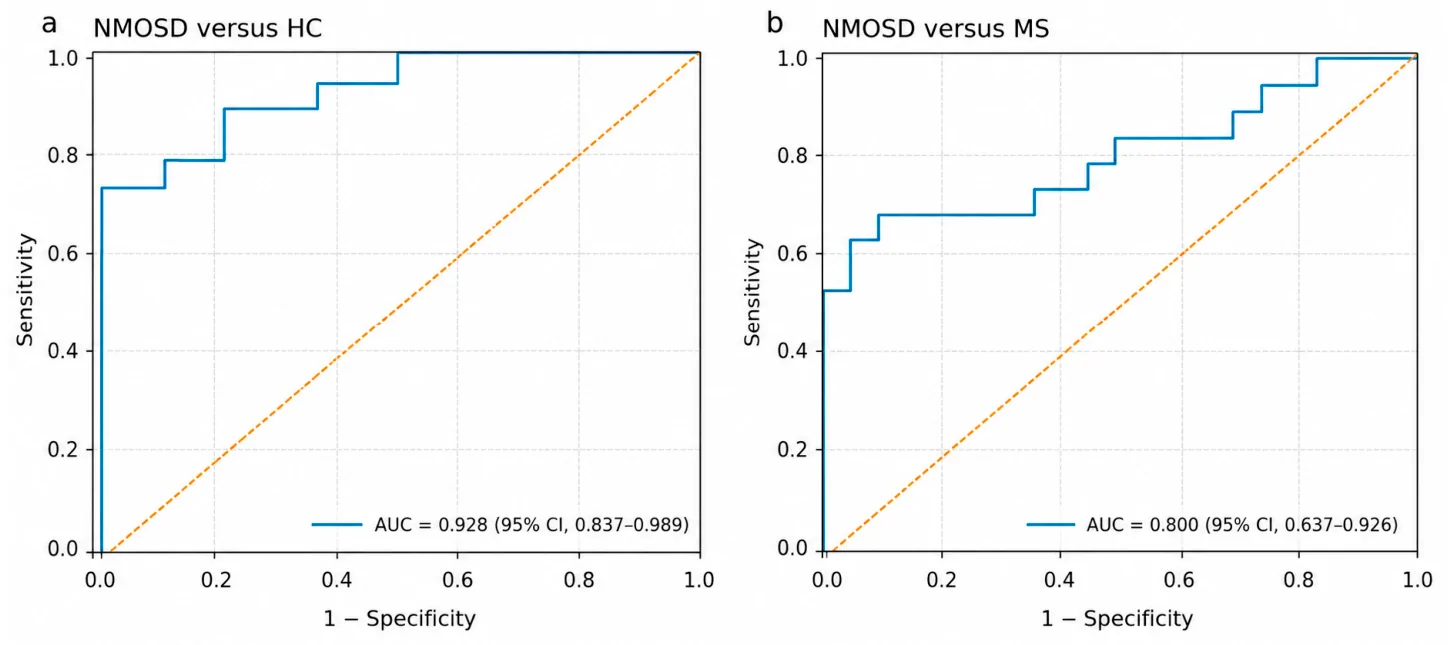
Receiver operating characteristic curves for serum SERPINA3 in the full ELISA cohort. (**a**) NMOSD versus HC (19 versus 19 participants). (**b**) NMOSD versus MS (19 versus 20 participants).

In the ELISA cohort, serum SERPINA3 distinguished NMOSD from HC with an AUC of 0.928 (95% CI, 0.837–0.989). At the Youden index-derived cutoff of 1661.99 μg/mL, sensitivity was 73.7% and specificity was 100.0%. For the clinically relevant distinction between NMOSD and MS, SERPINA3 yielded an AUC of 0.800 (95% CI, 0.637–0.926). A cutoff of 1845.56 μg/mL provided 68.4% sensitivity and 90.0% specificity (Figure 3 and Table 2).

In the post hoc sensitivity analysis excluding participants with preceding infection, 13 NMOSD, 19 MS, and 19 HC participants remained. SERPINA3 concentrations continued to differ across the three groups (Kruskal–Wallis H = 18.706, *p* = 8.67 × 10^−5^). Median concentrations were 2092.64 μg/mL (IQR, 1240.33–2568.53) in NMOSD, 1218.29 μg/mL (IQR, 645.98–1730.29) in MS, and 399.17 μg/mL (IQR, 227.11–807.11) in HC. In NMOSD-focused Dunn comparisons, the NMOSD-versus-HC difference remained significant after Holm adjustment (adjusted *p* = 4.13 × 10^−5^), whereas the NMOSD-versus-MS comparison was attenuated and no longer reached the conventional significance threshold (adjusted *p* = 0.0553). The corresponding AUCs were 0.895 (95% CI, 0.761–0.984) for NMOSD versus HC and 0.753 (95% CI, 0.547–0.923) for NMOSD versus MS (Table S4).

### 3.5. Routine Hematological Profiles and Correlations with SERPINA3

Routine hematological indices were analyzed in the complete-case subset (12 NMOSD, 12 MS, 14 TBI, and 19 HC). After Benjamini–Hochberg correction across 24 overall Kruskal–Wallis tests, 14 indices differed significantly among the four groups: WBC, lymphocyte count, monocyte count, neutrophil count, eosinophil count, basophil count, lymphocytes (%), neutrophils (%), eosinophils (%), basophils (%), RBC, MCV, MCH, and PDW. The strongest overall differences were observed for neutrophils (%) (H = 29.085, q = 5.05 × 10^−5^), lymphocytes (%) (H = 27.695, q = 5.05 × 10^−5^), neutrophil count (H = 26.284, q = 6.65 × 10^−5^), and WBC (H = 24.676, q = 0.000108). Post hoc Dunn tests with Holm adjustment indicated that most differences were driven by comparisons involving the TBI and HC groups. No routine hematological index differed significantly between NMOSD and MS after post hoc adjustment (Table 3 and Table S3).

**Figure 4:**
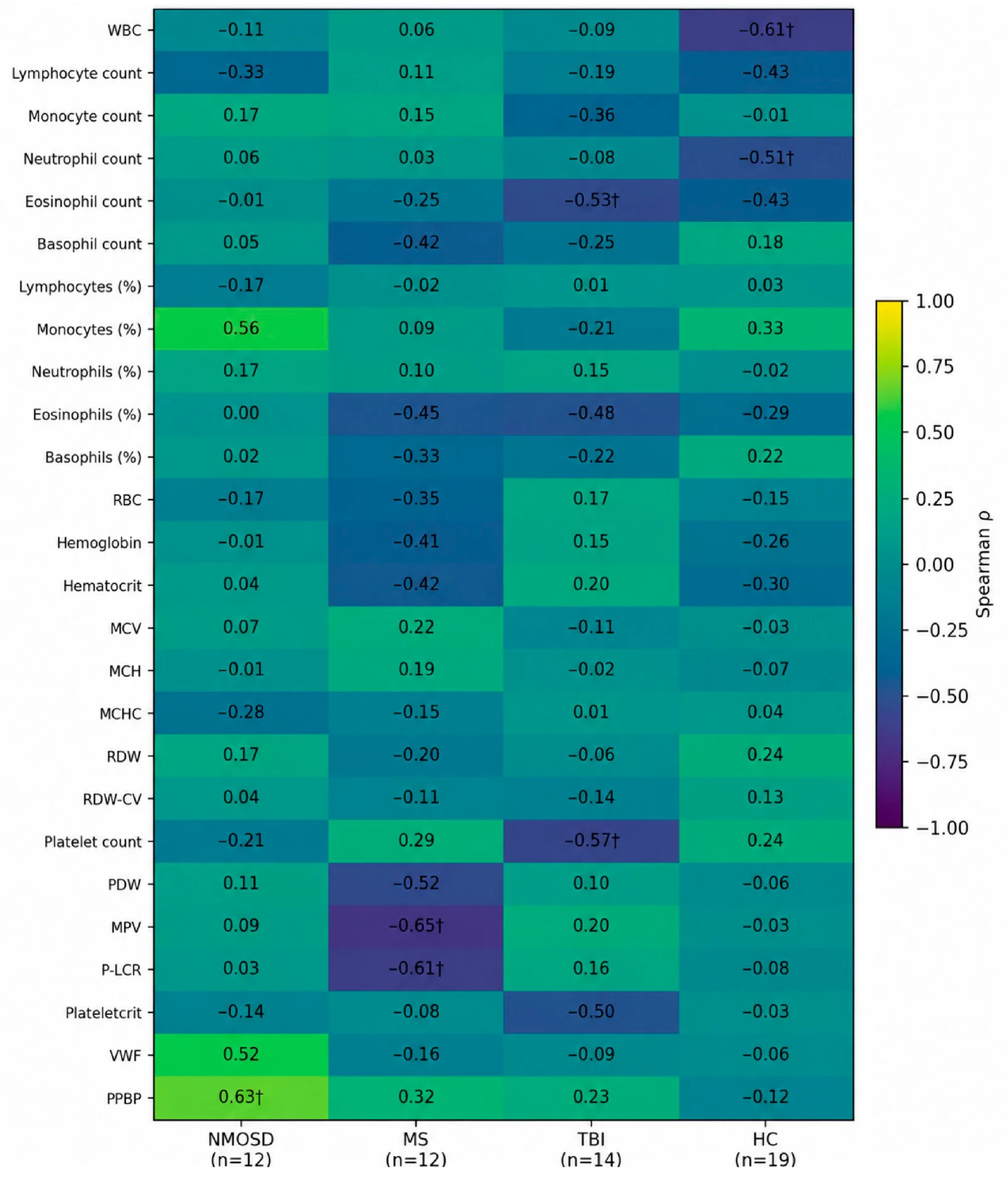
Group-specific correlations between serum SERPINA3 and other laboratory indicators. Values within the heatmap are Spearman correlation coefficients. The dagger indicates a nominal raw *p* < 0.05. Benjamini–Hochberg correction was applied separately across the 26 tested indicators within each group; no association remained significant at q < 0.05. HC, healthy controls; MS, multiple sclerosis; NMOSD, neuromyelitis optica spectrum disorder; TBI, traumatic brain injury.

Group-specific Spearman analyses identified nominal associations between SERPINA3 and other laboratory indicators: in NMOSD, PPBP (ρ = 0.629, raw *p* = 0.028); in MS, MPV (ρ = −0.649, raw *p* = 0.022) and P-LCR (ρ = −0.607, raw *p* = 0.036); in TBI, PLT (ρ = −0.572, raw *p* = 0.033) and eosinophil count (ρ = −0.533, raw *p* = 0.050); and in HC, WBC (ρ = −0.614, raw *p* = 0.005) and neutrophil count (ρ = −0.509, raw *p* = 0.026). None of these associations remained statistically significant after Benjamini–Hochberg correction across the 26 tests performed within each group (all q > 0.05) (Figure 4 and Table 4).

### 3.6. Diagnostic Performance of SERPINA3-Based Combined Models

For NMOSD versus HC in the complete-case subset (12 NMOSD and 19 HC), SERPINA3 alone yielded an AUC of 0.904 (95% CI, 0.776–0.991). Monocyte count was selected as the additional variable with the highest LOOCV performance. The SERPINA3–monocyte count model yielded an apparent AUC of 0.943 (95% CI, 0.851–1.000), with LOOCV AUCs of 0.846 for SERPINA3 alone and 0.904 for the combined model. The apparent AUCs did not differ significantly (DeLong *p* = 0.394).

**Figure 5:**
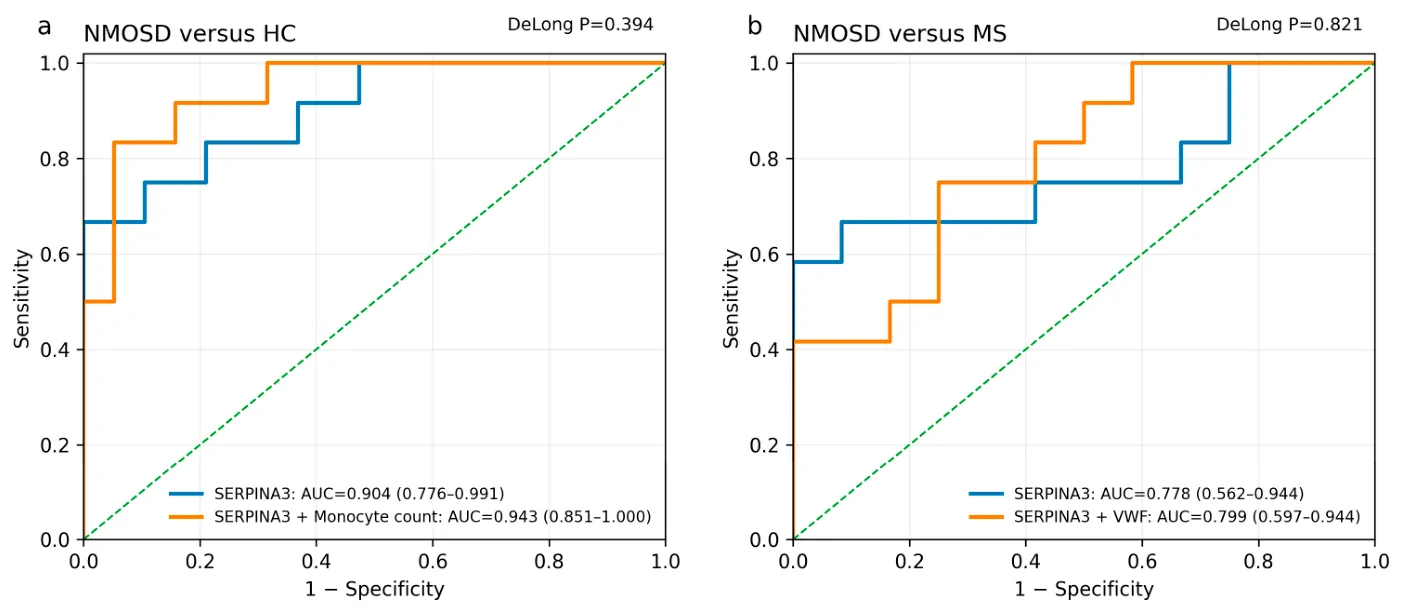
Receiver operating characteristic curves for serum SERPINA3 alone and the selected combined models in the complete-case subset. (**a**) NMOSD versus HC: SERPINA3 alone and SERPINA3 combined with monocyte count (12 patients with NMOSD and 19 HCs). (**b**) NMOSD versus MS: SERPINA3 alone and SERPINA3 combined with VWF (12 participants per group). AUC confidence intervals were estimated using 10,000 stratified bootstrap resamples, and correlated apparent AUCs were compared using the DeLong test. HC, healthy control; MS, multiple sclerosis; NMOSD, neuromyelitis optica spectrum disorder; VWF, von Willebrand factor.

For NMOSD versus MS in complete cases (12 participants per group), VWF was selected as the additional variable. SERPINA3 alone yielded an AUC of 0.778 (95% CI, 0.562–0.944), compared with 0.799 (95% CI, 0.597–0.944) for the SERPINA3–VWF model. The corresponding LOOCV AUCs were 0.688 and 0.701, respectively, with no significant difference between the apparent AUCs (DeLong *p* = 0.821) (Figure 5 and Table 5).

## 4. Discussion

This exploratory study combined TMT-based serum proteomic discovery with ELISA assessment in a separate, non-overlapping same-center cohort. The principal finding was a consistent increase in serum SERPINA3 in NMOSD, with moderate discriminatory performance for differentiating NMOSD from MS, whereas VWF and PPBP did not reproduce their discovery-phase signals by ELISA. These findings identify SERPINA3 as a preliminary adjunctive biomarker candidate that may complement established diagnostic evaluation rather than serve as a stand-alone diagnostic marker.

The discovery analysis identified 34 proteins meeting the prespecified fold-change criteria at nominal *p* < 0.05; however, none remained significant after correction for multiple testing across 261 proteins. Accordingly, the proteomic findings are best viewed as hypothesis-generating signals for candidate prioritization rather than as evidence of a stable proteomic signature or diagnostic classifier. Among the proteins showing higher abundance in NMOSD, VWF, PPBP, and SERPINA3 were selected for subsequent ELISA assessment on the basis of their discovery-phase signals and biological relevance.

ELISA assessment yielded different results for the three candidate proteins. VWF and PPBP did not show significant differences among the four groups, representing negative replication of their discovery-phase signals. Differences between the proteomic and ELISA findings may reflect differences in analytical platform, cohort composition, preanalytical handling, biological variability, or a combination of these factors. In contrast, SERPINA3 retained a consistent direction of change in the ELISA cohort, supporting its further evaluation as a candidate serum biomarker. To our knowledge, this is the first clinical study to specifically evaluate serum SERPINA3 as a candidate biomarker in NMOSD and to directly examine its ability to distinguish NMOSD from MS.

Previous studies have reported increased cerebrospinal fluid SERPINA3 in progressive MS, including an association with neurofilament light chain [22], while experimental and translational studies have implicated Serpina3n/SERPINA3 in CNS inflammation, tissue injury responses, and astrocyte-associated signaling [23,24,25]. SERPINA3 is an acute-phase serine protease inhibitor that can be produced systemically and expressed by reactive cells within the central nervous system [23]. Its elevation across multiple inflammatory and neurodegenerative conditions indicates that it is unlikely to be disease-specific. Nevertheless, the higher serum SERPINA3 concentrations observed in NMOSD than in MS may reflect differences in its magnitude of induction, cellular source, compartmental release, or temporal regulation. Given the astrocyte-centered pathology of AQP4-IgG-associated NMOSD [26,27,28] and reported associations between SERPINA3, reactive astrocytes, and neuroinflammatory responses [29,30], circulating SERPINA3 may reflect astrocytic injury, systemic acute-phase activation, protease regulation, or a combination of these processes. These observations provide a biological rationale for further investigation of SERPINA3 in NMOSD.

The sensitivity analysis excluding participants with preceding infection provides additional context for interpreting the SERPINA3 signal. The NMOSD–MS separation was attenuated after exclusion (0.800 vs. 0.753), whereas the overall group difference and the NMOSD-versus-HC comparison persisted. This pattern suggests that preceding infection may have contributed to the observed NMOSD–MS difference but does not fully explain the SERPINA3 findings.

The inclusion of both MS and TBI as neurological comparison groups further supports the clinical interpretation of the findings. MS represents the most clinically relevant inflammatory demyelinating alternative to NMOSD, whereas TBI provides a comparator for acute non-autoimmune central nervous system injury and physiological stress. The lower SERPINA3 concentrations observed in TBI than in NMOSD suggest that nonspecific neurological injury alone may not fully account for the elevated SERPINA3 levels observed in NMOSD.

In the full validation cohort, serum SERPINA3 showed moderate discrimination between NMOSD and MS (AUC: 0.800; sensitivity: 68.4%; specificity: 90.0%) and stronger separation between NMOSD and HCs (AUC: 0.928; sensitivity: 73.7%; specificity: 100%). Its sensitivity for the clinically relevant NMOSD–MS distinction was insufficient to support independent use as a screening or exclusion test. A more plausible clinical role is therefore as an adjunct to AQP4-IgG and MOG-IgG testing, neuroimaging, cerebrospinal fluid findings, and neurological assessment. Thus, the potential value of SERPINA3 lies in complementing rather than replacing established multimodal diagnostic evaluation.

Routine hematological analyses provided additional context for the SERPINA3 findings. Although several hematological indices differed overall among the four groups, none distinguished NMOSD from MS after corrected pairwise testing, and no correlation between SERPINA3 and the evaluated laboratory variables remained significant after within-group correction for multiple testing. In addition, the selected combined models did not significantly improve the AUC over SERPINA3 alone. These findings do not demonstrate a clear incremental diagnostic benefit from combining SERPINA3 with the evaluated routine hematological or protein markers.

This study has two main limitations. First, the clinical cohorts were relatively small and derived from a retrospective single-center population. The numbers of non-acute and AQP4-IgG-seronegative NMOSD cases and the complete-case subsets were limited, and some clinical and preanalytical information, including attack-to-sampling intervals, preceding infection, treatment exposure, processing intervals, fasting status, and sampling time, was incomplete. In addition, the comparator spectrum did not include a dedicated MOGAD group [31] or other inflammatory neurological disorders. These factors limited subgroup analyses, confounder adjustment, and generalizability. Second, the TMT-based proteomic discovery experiment had important design limitations [32]: diagnostic groups were assigned to fixed reporter channels, and no pooled bridge-reference channel was included. This design created structural confounding between disease group and reporter-channel effects and may have influenced the observed proteomic differences. The diagnostic performance and cutoff values reported here require confirmation in larger, prospective, multicenter cohorts with more complete clinical information, broader clinically relevant comparator groups, standardized preanalytical procedures, and balanced proteomic experimental designs.

## 5. Conclusions

Serum SERPINA3 showed potential value in supporting the differentiation of NMOSD from MS, but its current diagnostic performance is insufficient for use as a stand-alone test. It may provide complementary information within a multimodal diagnostic framework incorporating antibody testing, neuroimaging, cerebrospinal fluid findings, and clinical assessment. Prospective multicenter studies with clinically representative comparator groups, prespecified adjustment for relevant confounders, and independent external validation are required before clinical application.

## Figures and Tables

**Table 1:** Demographic and clinical characteristics of the validation cohort.

Characteristic	NMOSD (*n* = 19)	MS (*n* = 20)	TBI (*n* = 20)	HC (*n* = 19)	*p*
Gender (F/M)	16/3	16/4	15/5	16/3	1.000
Age at enrollment, years	33.1 (22, 44)	32.7 (23, 42)	36.8 (24, 50)	35.0 (23, 49)	0.463
Age at onset, years	31.3 (21, 41)	31.2 (23, 41)	-	-	0.979
Acute phase at sampling, *n* (%)	16 (84)	18 (90)	-	-	0.661
EDSS at onset	4.6 (3.1, 6.4)	2.8 (1.8, 3.5)	-	-	<0.001
AQP4-IgG ^a^, *n* (%)					
Seropositive	14 (73.7)	-	-	-	
Seronegative	5 (26.3); MOG-IgG ^a^: 1 positive, 4 negative	-	-	-	
Triggering factors, *n* (%)					
Fatigue	4 (21)	2 (10)	-	-	
Infection	6 (32)	1 (5)	-	-	0.036
Initial symptoms, *n* (%)					
Motor deficits	3 (16)	3 (15)	-	-	
Sensory deficits	6 (32)	10 (50)	-	-	
Visual disturbances	7 (37)	5 (25)	-	-	
Brainstem/cerebellar ^b^	9 (47)	5 (25)	-	-	
Higher-order dysfunction ^c^	0 (0)	3 (15)	-	-	
Other systemic ^d^	10 (53)	0 (0)	-	-	0.006

Values are median (IQR) unless otherwise indicated. *p* values for sex and age at enrollment compare all four groups; other *p* values compare NMOSD and MS. ^a^ Serum AQP4-IgG and MOG-IgG were both detected using cell-based assays (CBA). ^b^ includes unstable walking, headache/dizziness, nausea/vomiting; ^c^ includes consciousness disorder; ^d^ includes bladder/bowel dysfunction, hiccups, diplopia, tinnitus, hearing loss, respiratory distress, etc.; EDSS, Expanded Disability Status Scale; HC, healthy controls; MS, multiple sclerosis; NMOSD, neuromyelitis optica spectrum disorder; TBI, traumatic brain injury; “-’’ not applicable.

**Table 2:** Diagnostic performance of SERPINA3 in the full ELISA cohort.

Comparison	*n*	AUC (95% CI)	Cutoff	Sensitivity, %	Specificity, %
NMOSD vs. HC	19/19	0.928 (0.837–0.989)	1661.99 μg/mL	73.7 (48.8–90.9)	100.0 (82.4–100.0)
NMOSD vs. MS	19/20	0.800 (0.637–0.926)	1845.56 μg/mL	68.4 (43.4–87.4)	90.0 (68.3–98.8)

AUC confidence intervals were estimated using 10,000 stratified bootstrap resamples. Sensitivity and specificity confidence intervals are exact binomial intervals. Cutoffs were selected in the same dataset using the Youden index and require external validation. AUC, area under the receiver operating characteristic curve; HC, healthy controls; MS, multiple sclerosis; NMOSD, neuromyelitis optica spectrum disorder.

**Table 3:** Comparison of Routine Hematological Indices across the Four Groups.

Indicator	NMOSD (*n* = 12)	MS (*n* = 12)	TBI (*n* = 14)	HC (*n* = 19)	H	Raw *p*	BH q
WBC (×10^9^/L)	8.735 (4.980–9.890)	5.765 (4.902–7.808)	11.215 (10.143–13.838)	6.030 (5.360–6.945)	24.676	1.8 × 10^−5^	0.000108
LY# (×10^9^/L)	1.525 (1.157–2.015)	1.590 (0.897–1.867)	1.170 (0.870–1.535)	1.920 (1.720–2.355)	13.189	0.00425	0.00858
MONO# (×10^9^/L)	0.415 (0.338–0.595)	0.390 (0.328–0.465)	0.730 (0.477–0.917)	0.320 (0.260–0.360)	14.554	0.00224	0.00538
NEU# (×10^9^/L)	5.895 (3.115–7.577)	3.760 (3.012–5.145)	9.755 (8.755–12.510)	3.530 (3.155–4.460)	26.284	8.32 × 10^−6^	6.65 × 10^−5^
EO# (×10^9^/L)	0.020 (0.007–0.103)	0.075 (0.055–0.103)	0.010 (0.000–0.065)	0.130 (0.100–0.190)	15.967	0.00115	0.00346
BA# (×10^9^/L)	0.015 (0.010–0.022)	0.020 (0.010–0.022)	0.020 (0.010–0.030)	0.030 (0.025–0.050)	13.166	0.00429	0.00858
LY (%)	24.900 (17.575–33.175)	26.650 (14.175–30.700)	8.300 (6.375–10.675)	32.100 (26.300–38.200)	27.695	4.21 × 10^−6^	5.05 × 10^−5^
MONO (%)	6.500 (5.650–7.725)	6.300 (5.875–7.225)	5.100 (3.600–7.475)	5.200 (4.500–5.850)	8.008	0.0458	0.0688
NEU (%)	67.750 (59.800–78.325)	65.500 (61.525–75.125)	85.800 (79.250–89.475)	58.400 (54.750–64.650)	29.085	2.15 × 10^−6^	5.05 × 10^−5^
EO (%)	0.250 (0.000–2.100)	1.400 (0.900–1.800)	0.100 (0.000–0.475)	2.400 (1.350–3.150)	21.180	9.66 × 10^−5^	0.000464
BA (%)	0.100 (0.100–0.375)	0.350 (0.200–0.600)	0.150 (0.100–0.200)	0.600 (0.350–0.850)	18.722	0.000312	0.00125
RBC (×10^12^/L)	4.275 (3.853–4.412)	4.385 (4.077–4.478)	3.945 (3.540–4.230)	4.660 (4.405–4.870)	14.683	0.00211	0.00538
HGB (g/L)	126.000 (117.750–129.250)	124.000 (112.250–137.750)	126.500 (110.750–135.000)	134.000 (128.000–140.000)	6.453	0.0915	0.105
HCT (L/L)	0.375 (0.357–0.383)	0.365 (0.338–0.410)	0.375 (0.323–0.395)	0.400 (0.385–0.420)	8.491	0.0369	0.059
MCV (fL)	87.200 (85.475–91.250)	89.200 (82.150–90.850)	91.900 (89.350–94.350)	86.900 (85.450–88.800)	11.954	0.00754	0.0139
MCH (pg)	29.250 (28.475–30.125)	30.000 (27.375–30.800)	31.250 (30.550–32.175)	29.700 (28.050–30.000)	16.498	0.000896	0.00307
MCHC (g/L)	332.000 (329.750–335.750)	337.000 (328.000–341.250)	341.000 (336.500–348.750)	337.000 (330.000–341.000)	7.269	0.0638	0.0806
RDW (fL)	43.650 (41.675–45.325)	42.050 (41.000–42.250)	40.550 (39.875–41.275)	41.800 (41.150–43.200)	7.703	0.0526	0.0733
RDW-CV (%)	13.600 (12.925–14.300)	13.050 (12.550–14.350)	12.750 (12.200–13.250)	13.200 (12.850–13.500)	6.690	0.0825	0.099
PLT (×10^9^/L)	241.000 (219.000–317.250)	246.000 (222.500–274.000)	233.500 (171.250–298.000)	275.000 (250.000–314.500)	2.298	0.513	0.513
PDW (fL)	15.850 (15.500–16.200)	15.800 (15.600–16.000)	16.000 (15.900–16.175)	16.200 (15.950–16.400)	11.634	0.00875	0.015
MPV (fL)	8.950 (8.400–10.325)	9.650 (8.850–10.725)	8.950 (8.525–9.550)	9.900 (9.350–10.400)	6.263	0.0995	0.109
P-LCR (%)	19.400 (14.575–28.375)	24.700 (18.750–30.525)	19.200 (16.675–23.325)	25.800 (21.100–29.750)	6.012	0.111	0.116
PCT (%)	0.250 (0.202–0.275)	0.235 (0.228–0.258)	0.220 (0.152–0.260)	0.270 (0.250–0.295)	7.602	0.055	0.0733

Values are medians (interquartile ranges). Overall *p* values were obtained using the Kruskal–Wallis test. BH q values were calculated across the 24 routine hematological indices. WBC, white blood cell count; LY, lymphocyte; MONO, monocyte; NEU, neutrophil; EO, eosinophil; BA, basophil; RBC, red blood cell count; HGB, hemoglobin; HCT, hematocrit; MCV, mean corpuscular volume; MCH, mean corpuscular hemoglobin; MCHC, mean corpuscular hemoglobin concentration; RDW, red cell distribution width-standard deviation; RDW-CV, red cell distribution width-coefficient of variation; PLT, platelet count; PDW, platelet distribution width; MPV, mean platelet volume; P-LCR, platelet large-cell ratio; PCT, plateletcrit.

**Table 4:** Nominal Correlations between SERPINA3 and Other Indicators.

Group	Variable	*n*	Spearman ρ	Raw *p*	BH q
NMOSD	PPBP	12	0.629	0.028	0.694
MS	MPV	12	−0.649	0.022	0.472
MS	P-LCR	12	−0.607	0.036	0.472
TBI	Platelet count	14	−0.572	0.033	0.528
TBI	Eosinophil count	14	−0.533	0.050	0.528
HC	WBC	19	−0.614	0.005	0.134
HC	Neutrophil count	19	−0.509	0.026	0.339

Only correlations with raw *p* < 0.05 are shown. Benjamini–Hochberg correction was applied separately across 26 indicators within each diagnostic group.

**Table 5:** Diagnostic Performance of SERPINA3 Alone and the Selected Combined Models.

Comparison	Model	*n*	AUC (95% CI)	Cutoff	Se/Sp, %	LOOCV AUC	DeLong *p*
NMOSD vs. HC	SERPINA3	12/19	0.904 (0.776–0.991)	1869.92 μg/mL	66.7/100.0	0.846	Reference
NMOSD vs. HC	SERPINA3 + Monocyte count	12/19	0.943 (0.851–1.000)	0.475 probability	83.3/94.7	0.904	0.394
NMOSD vs. MS	SERPINA3	12/12	0.778 (0.562–0.944)	2092.64 μg/mL	58.3/100.0	0.688	Reference
NMOSD vs. MS	SERPINA3 + VWF	12/12	0.799 (0.597–0.944)	0.519 probability	75.0/75.0	0.701	0.821

Final fitted model equations (*p* denotes the predicted probability of NMOSD; z denotes standardization within the corresponding complete-case comparison dataset): NMOSD vs. HC: logit (*p*) = −0.090745 + 2.940852 × z (SERPINA3) + 2.933643 × z (Monocyte count), where z (SERPINA3) = [SERPINA3 − 1134.012258]/994.306488 and z (Monocyte count) = [Monocyte count − 0.395484]/0.174754. NMOSD vs. MS: logit (*p*) = −0.013527 + 1.533980 × z (SERPINA3) − 0.938679 × z (VWF), where z (SERPINA3) = [SERPINA3 − 1595.229583]/914.746394 and z (VWF) = [VWF − 46.089583]/21.613863. For both models, *p* = 1/[1 + exp (−logit (*p*))]. The equations represent the final models fitted to all available complete cases and are provided for reproducibility; they are not externally validated. AUC values are apparent estimates from models fitted and evaluated in the same complete-case data; LOOCV AUCs are exploratory internal estimates. SERPINA3-only cutoffs are concentrations in μg/mL, whereas combined-model cutoffs are unitless predicted probabilities selected using the Youden index. DeLong *p* values compare each selected combined model with SERPINA3 alone. The complete-case sample sizes were 12 NMOSD/19 HC and 12 NMOSD/12 MS; same-encounter routine laboratory data were unavailable for the remaining participants and were not imputed.

## Data Availability

The processed proteomic data underlying the reported analyses and associated analysis outputs are available from the corresponding author upon reasonable request.

## Supplementary Materials

The following supporting information can be downloaded at: https://www.mdpi.com/article/10.3390/diagnostics16162660/s1, Figure S1: Participant flow diagram for the discovery proteomic cohort. The discovery phase used an exploratory balanced sample set. Twenty NMOSD and 20 MS archived serum samples meeting the prespecified eligibility and analytical-quality criteria were selected for TMT-based proteomic analysis; Table S1: Baseline characteristics of the discovery proteomic cohort; Table S2: Exploratory differential-abundance analysis of 261 protein entries between the MS and NMOSD groups; Table S3: Significant post hoc comparisons for hematological indices; Table S4: Sensitivity analysis of serum SERPINA3 after excluding participants with preceding infection.

## Abbreviations

The following abbreviations are used in this manuscript:

NMOSD	Neuromyelitis optica spectrum disorder
MS	Multiple sclerosis
TBI	Traumatic brain injury
HC	Healthy control
EDSS	Expanded Disability Status Scale
TMT	Tandem mass tag
VWF	Von Willebrand factor
PPBP	Pro-platelet basic protein
SERPINA3	Serpin family A member 3
ELISA	Enzyme-linked immunosorbent assay
WBC	White blood cell
EO	Eosinophil
LY	Lymphocyte
NEU	Neutrophil
MONO	Monocyte
BA	Basophil
PDW	Platelet distribution width
PLT	Platelet
MPV	Mean platelet volume
P-LCR	Platelet large-cell ratio
PCT	Plateletcrit
AUC	Area under the curve
CI	Confidence interval
ROC	Receiver operating characteristic
AQP4	Aquaporin-4
CNS	Central nervous system
EAE	Experimental autoimmune encephalomyelitis
CSF	Cerebrospinal fluid
