# Supplementary material for: Serum SERPINA3 as a Candidate Non-Invasive Biomarker for Neuromyelitis Optica Spectrum Disorder: Proteomic Discovery and Same-Center Validation
Source: Diagnostics (Basel). 2026 Aug 20;16(16):2660. doi: 10.3390/diagnostics16162660 (PMC13511774; doi:10.3390/diagnostics16162660)
Supplement: Supplementary file 1 [file diagnostics-16-02660-s001.zip › diagnostics-4399390-supplementary.pdf]

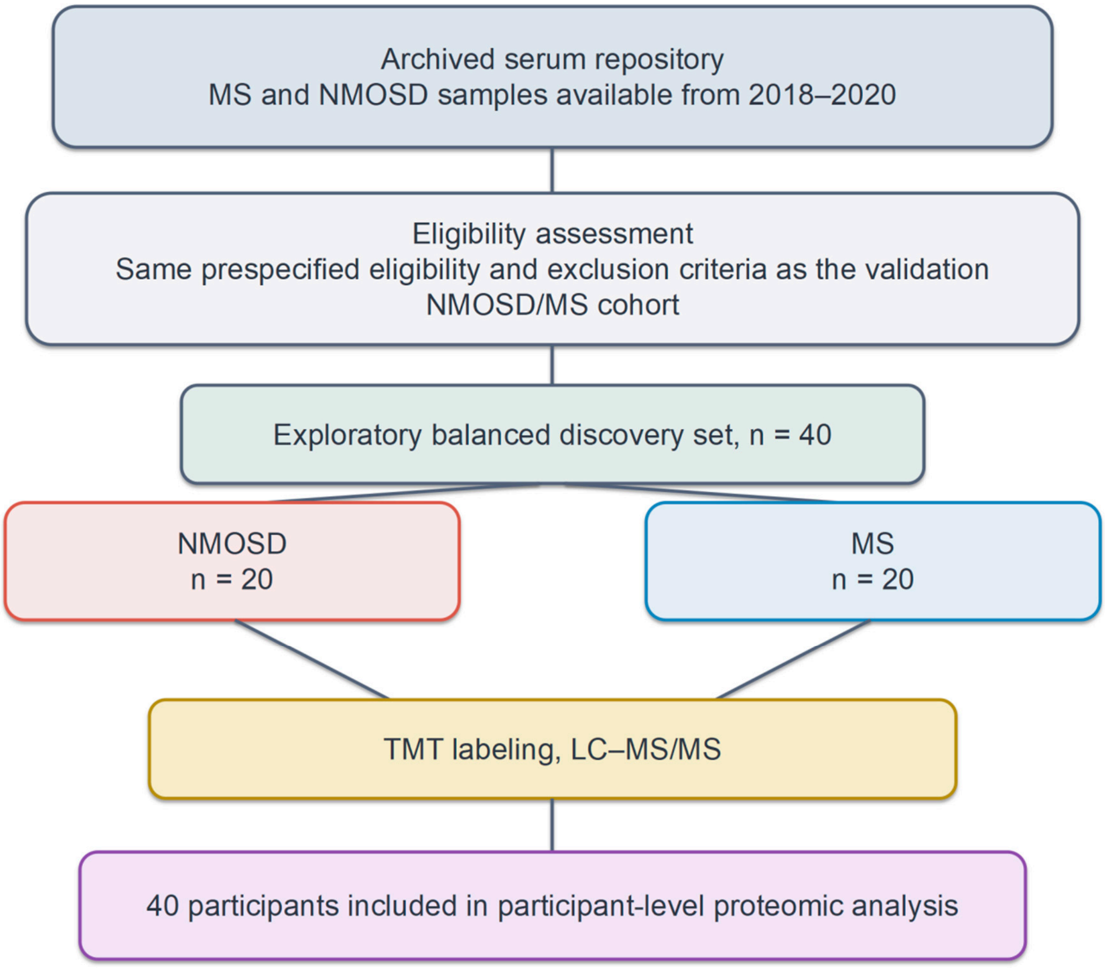

Figure S1. Participant flow diagram for the discovery proteomic cohort. The discovery phase used an exploratory balanced sample set. Twenty NMOSD and 20 MS archived serum samples meeting the prespecified eligibility and analytical-quality criteria were selected for TMT-based proteomic analysis.

Table S1. Baseline characteristics of the discovery proteomic cohort.

Characteristic	NMOSD (n=20)	MS (n=20)	P value
Gender (F/M)	17/3	16/4	1.000
Age at enrollment, years	33.0 (23.5, 40.3)	32.5 (25.0, 44.0)	0.516
Age at onset, years	30.5 (21.8, 38.0)	30.5 (23.0, 40.0)	0.655
EDSS at sampling	4.8 (3.8, 6.0)	2.5 (1.8, 3.2)	<0.001
Acute phase at sampling, n (%)	16 (80.0)	17 (85.0)	1.000
Disease subtype			
Relapsing NMOSD, n (%)	16 (80.0)	–	–
Monophasic NMOSD, n (%)	4 (20.0)	–	–
Relapsing-remitting MS, n (%)	–	17 (85.0)	–
Secondary progressive MS, n (%)	–	3 (15.0)	–

Values are median (IQR) unless otherwise indicated. Continuous variables were compared using the Mann–Whitney U test and categorical variables using Fisher's exact test. Disease-subtype categories are diagnosis-specific and were not compared inferentially. EDSS, Expanded Disability Status Scale; MS, multiple sclerosis; NMOSD, neuromyelitis optica spectrum disorder; "–", not applicable.

Table S2. Exploratory differential-abundance analysis of 261 protein entries between the MS and NMOSD groups.

Protein	MS/NMOSD	P value	Benjamini–Hochberg q value
ORM2	0.718	0.000281	0.073445
CFH	0.795	0.001107	0.129998
IGKV1D-33	0.661	0.002345	0.129998
CP	0.741	0.002736	0.129998
APOM	1.847	0.004534	0.129998
VWF	0.564	0.004682	0.129998
TTR	0.690	0.004965	0.129998
AMBP	0.785	0.005003	0.129998
Ig lambda chain V-II region BUR	1.614	0.005174	0.129998
IGLV1-36	0.411	0.005193	0.129998
Ig lambda chain V-I region HA	0.687	0.005526	0.129998
ATRN	1.644	0.006191	0.129998
PPBP	0.508	0.006747	0.129998
C1QC	1.503	0.007563	0.129998
IGLV8-61	3.249	0.008068	0.129998
PRG4	0.338	0.008183	0.129998
IGLV9-49	0.067	0.008467	0.129998
Ig lambda chain V-III region LOI	1.477	0.009215	0.131681
Ig heavy chain V-III region TIL	0.708	0.009586	0.131681
IGLV1-51	1.365	0.019762	0.243896
Ig heavy chain V-III region KOL	0.153	0.020549	0.243896
Ig lambda chain V-II region TRO	0.170	0.020558	0.243896
SERPINA3	0.623	0.021725	0.246537
IGKV3D-15	1.406	0.025942	0.271967
SERPINF2	1.329	0.026051	0.271967
Ig kappa chain V-II region MIL	0.161	0.027824	0.278847
C8G	0.750	0.029786	0.278847
FUT1	0.458	0.030792	0.278847
Ig heavy chain V-III region TUR;Ig heavy chain V-III region NIE	1.472	0.030983	0.278847
TMEM198	1.825	0.040140	0.338040
C1QB	1.219	0.040150	0.338040
IGKV1-5	0.724	0.043118	0.351681
BTD	0.147	0.044614	0.352853
LUM	1.248	0.046894	0.359983
HBD	2.851	0.051726	0.375744
ACTG1;ACTB;ACTG2	0.377	0.052274	0.375744
APOC2;APOC4-APOC2)	0.755	0.054506	0.375744
IGKV2D-29;IGKV2D-26	0.399	0.054706	0.375744
CFHR1	0.648	0.059499	0.397783
Ig heavy chain V-III region BUT	0.548	0.062266	0.397783
APOH	0.840	0.063377	0.397783
Ig kappa chain V-III region WOL;Ig kappa chain V-III region SIE	0.764	0.064739	0.397783
IGHV4-59;IGHV4-61;IGHV4-34;IGHV4-39	0.335	0.065535	0.397783
KIAA1217	0.404	0.068909	0.401605
PRDX	4.127	0.069242	0.401605
ORM1	0.727	0.076037	0.423274
IGLC7	0.248	0.076664	0.423274
SEPP1	0.589	0.078993	0.423274
Ig kappa chain V-I region BAN	0.544	0.079465	0.423274
SHBG	0.584	0.084965	0.443516
SERPINA4	1.205	0.088253	0.443967
IGHV3-30;IGHV3-23	1.332	0.088453	0.443967
CFP	1.349	0.090182	0.444102
CLU	0.791	0.099569	0.469317
Ig lambda chain V-IV region Hil	0.783	0.102496	0.469317
APOB	0.891	0.103157	0.469317
Ig kappa chain V-I region Roy	1.413	0.104200	0.469317
GPLD1	1.383	0.104293	0.469317
IGKV3-11;IGKC	0.857	0.111324	0.492469
GC	4.251	0.113708	0.494632
IGLV3-19	0.630	0.118214	0.498023
CFD	0.170	0.119137	0.498023
IGFBP3	1.367	0.121896	0.498023
LPA	2.060	0.122227	0.498023
APOC1	0.768	0.124029	0.498023
APOE	0.836	0.130143	0.514655
CA2	4.832	0.135794	0.522645
FCN3	0.862	0.136168	0.522645
Ig kappa chain V-I region DEE	2.840	0.140936	0.530492
FBLN1	2.399	0.142277	0.530492
FCN2	4.928	0.145897	0.534620

Protein	MS/NMOSD	P value	Benjamini–Hochberg q value
GSN	0.814	0.147481	0.534620
APCS	0.595	0.152288	0.544482
Ig kappa chain V-I region Ni	1.698	0.165092	0.582286
TOR3A	0.833	0.174947	0.608816
LCAT	4.498	0.178936	0.614011
PROC	1.239	0.181967	0.614011
CPN1	0.798	0.186502	0.614011
IGLL5;IGLC1	1.275	0.188501	0.614011
MASP2	0.601	0.190393	0.614011
C7	0.834	0.192501	0.614011
APOD	0.807	0.192908	0.614011
PLTP	0.146	0.195675	0.614210
IGHA2	0.694	0.197677	0.614210
IGHG1	10.105	0.204157	0.626882
IGLV1-47;IGLV1-44	1.632	0.208962	0.627758
IGKV3D-20	1.457	0.213721	0.627758
AASDH	0.114	0.215598	0.627758
KLKB1	0.801	0.223859	0.627758
IGLV3-10	1.290	0.228623	0.627758
Ig kappa chain V-I region Kue	0.502	0.229234	0.627758
SAA1	0.434	0.229610	0.627758
FGA	1.368	0.233438	0.627758
LGALS3BP	0.692	0.233924	0.627758
SERPINA1	0.901	0.235105	0.627758
HGFAC	0.193	0.236903	0.627758
IGHG2	0.849	0.239860	0.627758
IGHV3-49	1.252	0.241330	0.627758
IGKV4-1	0.834	0.242037	0.627758
IGKV3-20	0.262	0.243547	0.627758
Ig lambda chain V-I region WAH	0.655	0.246513	0.627758
SERPING1	1.192	0.247857	0.627758
Ig kappa chain V-III region GOL	2.647	0.248236	0.627758
C1R	1.097	0.250141	0.627758
AGT	0.862	0.269293	0.659439
GC	1.096	0.273659	0.659439
IGHV3-72;IGHV3-73;IGHV3-15	1.149	0.281235	0.659439
IGHG1	0.884	0.282347	0.659439
Ig heavy chain V-III region JON	1.548	0.287299	0.659439
IGHV5-51	1.227	0.290016	0.659439
IGHV3OR16-12	0.770	0.291722	0.659439
IGLV3-9	0.121	0.291778	0.659439
TTC30B;TTC30A	1.894	0.292679	0.659439
SSFA2	3.388	0.303880	0.659439
IGLV2-18	0.767	0.308581	0.659439
IGLV3-16	0.785	0.310798	0.659439
HBB	0.821	0.311568	0.659439
ZNF814	2.895	0.311782	0.659439
VTN	0.890	0.311877	0.659439
IGHG1	0.687	0.313260	0.659439
C8B	1.189	0.317963	0.659439
HRG	0.855	0.318179	0.659439
ALB	0.836	0.320933	0.659439
HP	0.854	0.320967	0.659439
C4BPB	1.135	0.324957	0.659439
KNG1	0.451	0.327242	0.659439
GPX3	1.194	0.327958	0.659439
SFPQ	0.011	0.328886	0.659439
LAMP2	2.533	0.329385	0.659439
IGLL1	1.876	0.329476	0.659439
ITIH3	0.816	0.330983	0.659439
IGLV2-8	1.203	0.338235	0.661735
GP1BA	0.791	0.339422	0.661735
C4BPA	1.078	0.341462	0.661735
HSPG2	0.131	0.342277	0.661735
C8A	0.909	0.345383	0.662831
APOA2	1.145	0.349052	0.663913
SERPINA10	0.826	0.351034	0.663913
A2M	0.901	0.371657	0.680370
F10	0.879	0.380767	0.680370
IGKV3-15;IGKV3OR2-268;IGKV3D-7;IGKV3-7	1.190	0.382377	0.680370
ECM1	0.877	0.383653	0.680370
HPX	0.925	0.384520	0.680370
CD5L	0.894	0.386418	0.680370
SAA2-SAA4;SAA4	1.145	0.386643	0.680370
C2	0.721	0.386959	0.680370
SERPINF1	0.870	0.387553	0.680370
Ig kappa chain V-III region NG9	1.162	0.387688	0.680370
KNG1	0.936	0.388411	0.680370
IGKV2D-28;IGKV2-40;IGKV2D-40	0.879	0.392510	0.682968
LRG1	0.840	0.397391	0.685123
APOA1	0.923	0.400182	0.685123
C4A	1.244	0.401734	0.685123
FCGR3A	0.658	0.404249	0.685123
CPN2	0.867	0.419400	0.699005

Protein	MS/NMOSD	P value	Benjamini–Hochberg q value
ITIH1	0.929	0.419801	0.699005
CFI	0.937	0.423239	0.699005
IGKV1-8	0.696	0.424720	0.699005
C5	1.060	0.425831	0.699005
IGJ;JCHAIN	1.127	0.430277	0.699052
F9	0.692	0.431216	0.699052
ITIH4	1.074	0.437553	0.701208
NDUFB3	0.263	0.439790	0.701208
KRT2	0.617	0.444664	0.701208
TF	0.933	0.445485	0.701208
FN1	1.075	0.445979	0.701208
MDN1	0.712	0.458259	0.714130
SELL	1.376	0.459670	0.714130
IGLV2-11	1.209	0.467189	0.721517
MST1	0.739	0.483343	0.728357
PON1	1.071	0.486783	0.728357
APOA4	1.087	0.487117	0.728357
SPARC	0.485	0.487808	0.728357
CDC5L	2.303	0.492329	0.728357
DEFA3;DEFA1	1.288	0.496627	0.728357
F5	1.191	0.497284	0.728357
C1RL	0.897	0.498513	0.728357
IGHA2	1.153	0.500843	0.728357
FBLN1	1.262	0.501339	0.728357
HPR	1.160	0.502315	0.728357
Ig kappa chain V-I region Wes	0.720	0.506665	0.730605
FCGBP	0.687	0.512178	0.734496
IGHG3	0.910	0.520974	0.738060
SERPIND1	0.917	0.522449	0.738060
C4B	1.049	0.523901	0.738060
Ig heavy chain V-I region V35	1.115	0.530015	0.738060
AHSG	1.154	0.530765	0.738060
S100A9	0.614	0.531630	0.738060
HABP2	0.825	0.540292	0.746117
APOL1	1.125	0.544893	0.748511
KRT9	1.937	0.557669	0.762050
CPB2	0.756	0.561650	0.763492
PF4;PF4V1	0.888	0.587623	0.789051
F12	1.106	0.589396	0.789051
THBS1	0.919	0.591456	0.789051
APOC4	0.903	0.592544	0.789051
TGFBI	1.434	0.604824	0.801315
TIAM1	1.207	0.612918	0.807937
IGLV2-23	0.766	0.619132	0.811246
AZGP1	0.939	0.621645	0.811246
IGHG1	0.740	0.633646	0.822795
IGHG4	0.938	0.642917	0.830699
IGHD	0.866	0.655811	0.841035
PROS1	0.957	0.657360	0.841035
PGLYRP2	0.955	0.670092	0.849989
C1QA	1.097	0.673498	0.849989
PLG	0.963	0.674129	0.849989
APMAP	1.165	0.683122	0.857187
ICAM2	1.263	0.693440	0.858590
PZP	0.860	0.694290	0.858590
OAF	1.331	0.701552	0.858590
IGHV4-28	1.100	0.702002	0.858590
IGKV2-24;IGKV2D-24	0.751	0.704469	0.858590
Ig lambda chain V-II region NIG-84	0.946	0.705559	0.858590
KRT10	0.864	0.707267	0.858590
MBD3L5	0.721	0.713658	0.862336
IGHM	0.962	0.722872	0.869445
BCHE	0.930	0.743532	0.890192
IDE	1.057	0.754820	0.892567
IGHV3-35	1.221	0.754990	0.892567
A1BG	0.974	0.755775	0.892567
FETUB	1.048	0.764978	0.894758
IGLC3;IGLC2	1.026	0.766578	0.894758
IGHM	1.081	0.767915	0.894758
Ig heavy chain V-II region ARH-77	1.034	0.773199	0.896911
IFITM3	1.124	0.789453	0.899974
SERPINA6	1.041	0.790723	0.899974
IGFALS	0.937	0.792552	0.899974
IGHV3-13	0.891	0.792898	0.899974
APOC3	0.967	0.796191	0.899974
C6	0.969	0.796528	0.899974
C1S	0.971	0.810758	0.905157
APOF	1.160	0.811831	0.905157
CA1	1.038	0.814926	0.905157
CST3	1.286	0.814988	0.905157
IGLV2-14	1.181	0.837836	0.926590
IGF2	0.898	0.841759	0.927001
IGLV7-43;IGLV7-46	1.070	0.852737	0.935144
F13B	0.956	0.866515	0.944769

Protein	MS/NMOSD	P value	Benjamini–Hochberg q value
LRRK2	0.947	0.868753	0.944769
AFM	1.018	0.874727	0.947318
MEGF8	1.106	0.882369	0.951646
SERPINC1	0.987	0.886137	0.951777
C9	0.989	0.913994	0.977673
IGHA1	0.981	0.918917	0.978643
HBA1	1.023	0.924832	0.978643
ALDOC	0.966	0.926148	0.978643
FAT4	0.953	0.939068	0.988294
LBP	0.986	0.953817	0.989651
CA5B	0.984	0.954310	0.989651
C3	0.997	0.957110	0.989651
F2	1.003	0.964781	0.989651
SHISA6	1.021	0.965626	0.989651
CLEC3B	0.994	0.967978	0.989651
IGKV3D-11	0.993	0.971567	0.989651
SERPINA7	0.996	0.972765	0.989651
CFB	1.002	0.977285	0.989651
RBP4	0.997	0.983644	0.989651
ITIH2	0.999	0.984385	0.989651
CNDP1	0.997	0.985859	0.989651
IGKV1-6;IGKV1-17	1.007	0.991315	0.991315

Fold change is expressed as MS/NMOSD. Nominal P values were obtained from independent two-sample t tests, and q values were calculated using the Benjamini–Hochberg procedure across all 261 protein tests. Exploratory candidate prioritization required nominal P < 0.05 together with fold change > 1.2 or < 1/1.2. Thirty-four protein entries met these nominal criteria; none achieved q < 0.05.

Table S3. Significant post hoc comparisons for hematological indices

Indicator	Comparison	Direction	Z	Raw P	Holm-adjusted P
WBC ($\times 10^9$/L)	NMOSD vs TBI	NMOSD < TBI	-2.912	0.0036	0.0144
WBC ($\times 10^9$/L)	MS vs TBI	MS < TBI	-3.971	7.16e-05	0.000358
WBC ($\times 10^9$/L)	TBI vs HC	TBI > HC	4.602	4.19e-06	2.51e-05
LY# ($\times 10^9$/L)	TBI vs HC	TBI < HC	-3.558	0.000374	0.00225
MONO# ($\times 10^9$/L)	TBI vs HC	TBI > HC	3.709	0.000208	0.00125
NEU# ($\times 10^9$/L)	NMOSD vs TBI	NMOSD < TBI	-2.963	0.00305	0.0122
NEU# ($\times 10^9$/L)	MS vs TBI	MS < TBI	-3.958	7.55e-05	0.000378
NEU# ($\times 10^9$/L)	TBI vs HC	TBI > HC	4.831	1.36e-06	8.14e-06
EO# ($\times 10^9$/L)	NMOSD vs HC	NMOSD < HC	-2.919	0.00351	0.0176
EO# ($\times 10^9$/L)	TBI vs HC	TBI < HC	-3.678	0.000235	0.00141
BA# ($\times 10^9$/L)	NMOSD vs HC	NMOSD < HC	-3.111	0.00187	0.0112
BA# ($\times 10^9$/L)	MS vs HC	MS < HC	-2.852	0.00434	0.0217
LY (%)	NMOSD vs TBI	NMOSD > TBI	3.055	0.00225	0.0113
LY (%)	MS vs TBI	MS > TBI	3.029	0.00245	0.0113
LY (%)	TBI vs HC	TBI < HC	-5.239	1.62e-07	9.7e-07
NEU (%)	NMOSD vs TBI	NMOSD < TBI	-3.121	0.00181	0.00903
NEU (%)	MS vs TBI	MS < TBI	-3.089	0.00201	0.00903
NEU (%)	TBI vs HC	TBI > HC	5.370	7.86e-08	4.71e-07
EO (%)	NMOSD vs HC	NMOSD < HC	-2.693	0.00708	0.0354
EO (%)	TBI vs HC	TBI < HC	-4.502	6.74e-06	4.04e-05
BA (%)	NMOSD vs HC	NMOSD < HC	-3.080	0.00207	0.0103
BA (%)	TBI vs HC	TBI < HC	-4.004	6.24e-05	0.000374
RBC ($\times 10^{12}$/L)	NMOSD vs HC	NMOSD < HC	-2.667	0.00765	0.0382
RBC ($\times 10^{12}$/L)	TBI vs HC	TBI < HC	-3.596	0.000324	0.00194
MCV (fL)	TBI vs HC	TBI > HC	3.352	0.000803	0.00482
MCH (pg)	NMOSD vs TBI	NMOSD < TBI	-3.097	0.00195	0.00976
MCH (pg)	MS vs TBI	MS < TBI	-2.810	0.00495	0.0198
MCH (pg)	TBI vs HC	TBI > HC	3.773	0.000162	0.000969
PDW (fL)	MS vs HC	MS < HC	-3.174	0.0015	0.00901

Only pairwise comparisons with Holm-adjusted P < 0.05 are shown. Dunn tests were performed only for indices with an overall Benjamini–Hochberg q < 0.05. WBC, white blood cell count; LY, lymphocyte; MONO, monocyte; NEU, neutrophil; EO, eosinophil; BA, basophil; RBC, red blood cell count; MCV, mean corpuscular volume; MCH, mean corpuscular hemoglobin; PDW, platelet distribution width.

Table S4. Sensitivity analysis of serum SERPINA3 after excluding participants with preceding infection.

Panel A. SERPINA3 concentrations after exclusion of preceding infection.

Group	n	SERPINA3, median (IQR), μg/mL

Group	n	SERPINA3, median (IQR), μg/mL
NMOSD	13	2092.64 (1240.33–2568.53)
MS	19	1218.29 (645.98–1730.29)
HC	19	399.17 (227.11–807.11)

Overall Kruskal–Wallis test: $H = 18.706$, $P = 8.67 \times 10^{-5}$.

Panel B. NMOSD-focused pairwise comparisons and diagnostic performance.

Comparison	Dunn z	Raw P	Holm-adjusted P	AUC (95% CI)
NMOSD vs HC	4.258	2.07×10^{-5}	4.13×10^{-5}	0.895 (0.761–0.984)
NMOSD vs MS	1.917	0.0553	0.0553	0.753 (0.547–0.923)

Six NMOSD and one MS participant with a clinically documented preceding infection were excluded. The single AQP4-IgG-seronegative/MOG-IgG-positive participant was among the NMOSD participants excluded because of preceding infection. Pairwise comparisons were performed using Dunn tests with Holm adjustment for the two NMOSD-focused comparisons. AUC 95% confidence intervals were estimated using 10,000 stratified bootstrap resamples. The sensitivity analysis was post hoc.
